# Immunological barriers and engineering strategies for CAR-T cell therapy in acute myeloid leukemia

**DOI:** 10.3389/fimmu.2026.1837609

**Published:** 2026-07-15

**Authors:** Ya Wang, Tiantian Yu, Ming Wang, Li Yu

**Affiliations:** Department of Hematology, The Second Affiliated Hospital, Jiangxi Medical College, Nanchang University, Jiangxi Provincial Key Laboratory of Hematological Diseases (2024SSY06052), Nanchang, Jiangxi, China

**Keywords:** acute myeloid leukemia, bone marrow microenvironment, CAR-T cell therapy, immunosuppression, leukemic heterogeneity, target specificity

## Abstract

Acute myeloid leukemia (AML) remains a difficult disease to treat, especially in patients with relapsed or refractory disease. While chimeric antigen receptor T cell (CAR-T) therapy has transformed the treatment landscape of several B-cell malignancies, its clinical efficacy in AML has been substantially more limited. This limited efficacy reflects not only challenges in CAR design, but also the complex biological and immunological barriers inherent to AML. Insufficient target specificity, pronounced leukemic heterogeneity, and an immunosuppressive bone marrow microenvironment collectively impair CAR-T cell recognition, persistence, and effector function, thereby restricting both therapeutic efficacy and safety. In this review, we discuss these major barriers and summarize emerging engineering strategies developed to address them, including approaches to improve targeting precision, reinforce CAR-T cell functional fitness, and remodel the suppressive immune niche. Together, these insights may help clarify the key barriers to effective CAR-T therapy in AML and inform future strategies for its optimization.

## 1 Introduction

Acute myeloid leukemia (AML) is a highly heterogeneous and aggressive hematologic malignancy that remains difficult to treat, particularly in the relapsed or refractory setting (1, 2). In recent years, treatment options for AML have expanded considerably, with improvements in conventional chemotherapy, the clinical introduction of targeted agents, as well as continuous improvements in allogeneic hematopoietic stem cell transplantation (allo-HSCT) (3, 4). Nevertheless, a large proportion of patients still experience relapse or develop refractory disease, with persistently poor long-term survival. Chimeric antigen receptor T cell (CAR-T) therapy, a form of adoptive immunotherapy in which T cells are genetically engineered to recognize cell-surface antigens, has achieved remarkable success in relapsed or refractory B-cell malignancies and multiple myeloma, providing a strong rationale for extending this strategy to AML (5–8).

However, the application of CAR-T cell therapy in AML has been far more challenging. Unlike B cell malignancies, in which lineage-associated antigens such as CD19 can be targeted with manageable toxicity, AML lacks ideal target antigens with sufficient leukemia specificity, exhibits pronounced inter- and intrapatient heterogeneity, and develops within a highly immunosuppressive bone marrow microenvironment that undermines CAR-T cell persistence and effector function. These AML-specific biological and immunological features limit both therapeutic efficacy and safety and have slowed clinical translation. In this review, we discuss the major barriers that restrict CAR-T therapy in AML and summarize emerging engineering strategies designed to improve targeting precision, preserve CAR-T cell functional fitness, and overcome microenvironmental suppression.

## 2 Overview of CAR-T cell therapy

The incorporation of costimulatory domains such as CD28 or 4-1BB established second-generation CARs as the dominant clinical platform (9–13). Subsequent designs incorporated additional regulatory features, including cytokine secretion in armored CAR-T cells and cytokine receptor signaling modules such as JAK-STAT pathways, to further enhance functional persistence (14–18), as illustrated in **Supplementary Figure 1** These developments are particularly relevant in AML, where sustained activity and adaptation to the marrow microenvironment are critical determinants of therapeutic efficacy.

## 3 Major biological and immunological barriers to CAR-T therapy in AML

The biological and immunological features of AML create barriers that are fundamentally different from those in B cell malignancies and have markedly slowed the clinical development of CAR-T therapy in this disease. These challenges are highly interconnected and collectively limit both the efficacy and safety of CAR-T therapy in AML. Broadly, these challenges can be grouped into three major categories: target-related constraints, leukemic heterogeneity, and the immunosuppressive marrow microenvironment.

### 3.1 Target antigen limitations in AML

**Figure 1:**
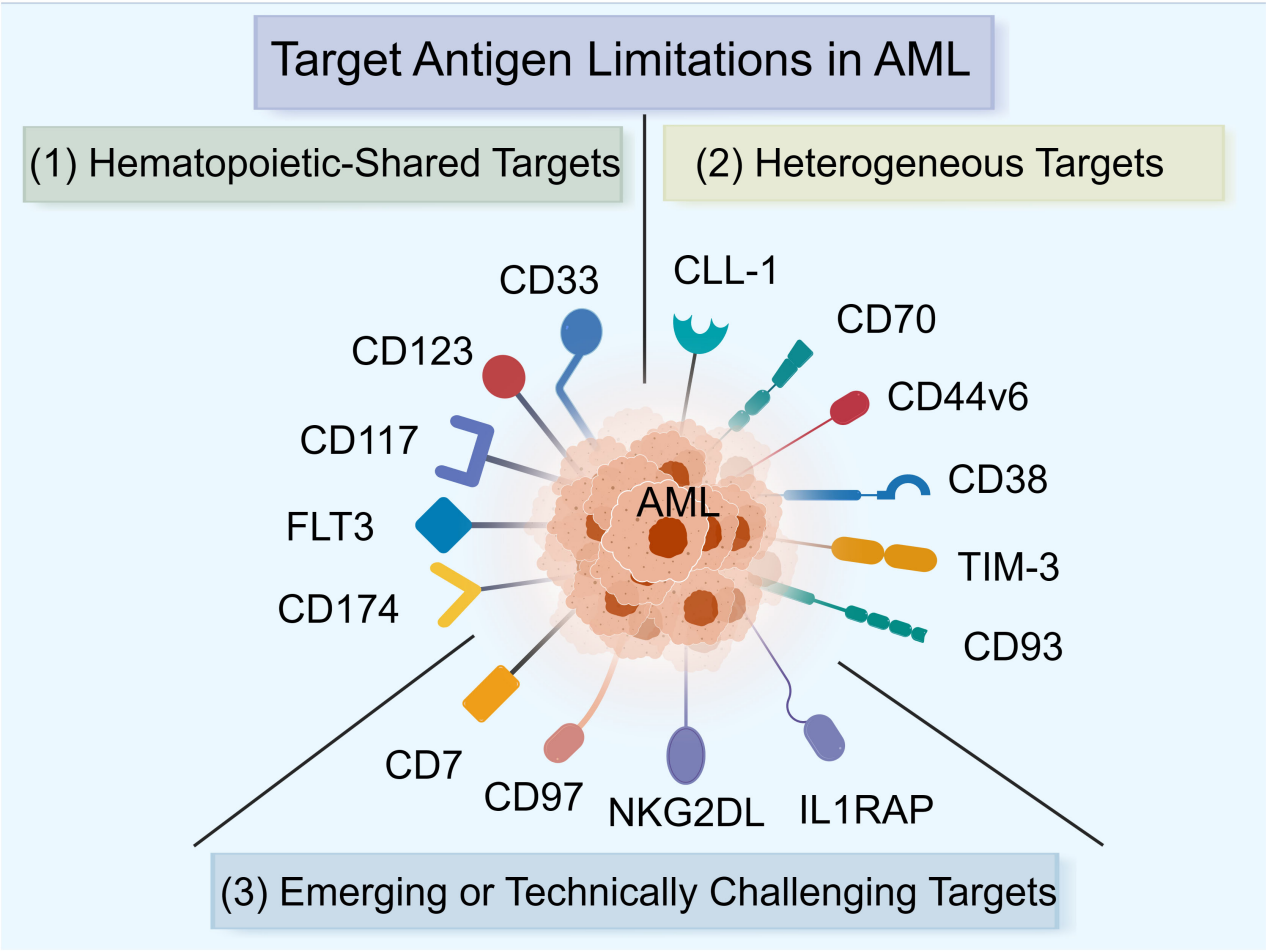
Major categories of target antigen limitations in AML. Target antigens for AML CAR-T therapy can be broadly classified into three groups: (1) hematopoietic-shared targets, which raise concerns about on-target/off-tumor toxicity. (2) heterogeneous targets, which may contribute to incomplete leukemic coverage and antigen escape. (3) emerging or technically challenging targets, which offer additional opportunities but remain limited by biological and translational barriers.

Target selection remains the central obstacle in AML CAR-T therapy. An ideal target would be uniformly and stably expressed on AML blasts, particularly leukemic stem cells (LSCs), while being absent from normal hematopoietic stem and progenitor cells (HSPCs) and other essential tissues (19, 20). In AML, however, this condition is rarely met. Because AML arises within the hematopoietic system, many candidate antigens are shared with normal myeloid development, leaving only a narrow therapeutic window (21). Clinically, this remains the defining bottleneck of the field. Based on the degree of overlap with normal hematopoietic stem cells (HSCs), currently explored targets can be broadly classified into three categories, all of which exhibit significant limitations (**Figure 1**).

#### 3.1.1 Targets with broad overlap with normal hematopoiesis

CD33 and CD123 best illustrate this problem. Both are highly expressed on AML blasts and LSCs, but they are also present on normal HSPCs and mature myeloid cells (22). As a result, CAR-T engagement can damage normal hematopoiesis as well as leukemia, leading to prolonged cytopenias, transfusion dependence, and increased risks of infection and bleeding (23, 24). This toxicity profile sharply limits the therapeutic window and may also compromise subsequent consolidation, including transplantation, representing one of the major translational challenges in AML CAR-T therapy (25). Similar concerns apply to CD117, FLT3, and Lewis Y (CD174), which likewise carry a substantial risk of marrow aplasia because of their expression on normal hematopoietic cells (26, 27).

These risks are not confined to the marrow. Expression in non-hematopoietic tissues may give rise to organ-specific toxicities. CD123 expression on endothelial cells has been linked to capillary leak syndrome, while CD33 expression on hepatic sinusoidal Kupffer cells has been associated with sinusoidal obstruction syndrome (24, 28). Given their narrow therapeutic windows, these broadly expressed targets are clinically the most difficult to deploy as standalone therapies; their most realistic niche is in bridge-to-transplant settings or as components of safety-controlled or dual-targeting platforms.

#### 3.1.2 Relatively restricted targets limited by heterogeneity

To widen the therapeutic window, increasing attention has shifted toward antigens that are absent or minimally expressed on normal HSCs but enriched on AML cells, including LSCs. This is a necessary step forward, but it does not fully resolve the problem. Although these relatively lineage-restricted targets may reduce direct hematopoietic toxicity, they are still associated with important clinical limitations.

CLL-1 (CLEC12A) has emerged as a prominent example. It is highly expressed on AML blasts and LSCs, while largely absent on normal HSCs and minimally expressed in non-myeloid tissues, suggesting a more favorable safety profile than CD33 or CD123 (29–33). However, because CLL-1 is still expressed on mature neutrophils and monocytes, CLL-1-directed CAR-T therapy has remained associated with substantial myelosuppression and severe pancytopenia in clinical settings (34). Nevertheless, among currently explored single targets, CLL-1 has shown some of the most robust objective response rates and is arguably the most clinically mature antigen in AML CAR-T development, although durable disease control remains limited by antigen escape and T cell persistence.

CD70 presents a different type of limitation. Although it is expressed on a subset of AML blasts and is absent on HSCs (35), its expression varies markedly among patients, with reported positivity rates ranging from about 30% to nearly 100% (36). Because CAR-T activity often depends on antigen density, this variability reduces both the predictability and generalizability of CD70 as a single target (37).

Other candidates, including CD44v6, CD38, TIM-3, and CD93, face similar challenges (38–43). The major challenge with CD70 is therefore not safety but target stability, highlighting antigen heterogeneity rather than lineage overlap as a dominant failure mode in this category. Of these, CD38 and CD70 have entered early-phase trials, but their evidence maturity remains substantially lower than that of CLL-1.

#### 3.1.3 Emerging targets constrained by biological or technical trade-offs

With continued advances in AML biology, several emerging targets characterized by distinct biological features or specific technical challenges have gained attention. CD7 is aberrantly expressed in 30%-40% of AML cases but is absent in normal myeloid cells, suggesting relative tumor selectivity (44). However, its expression on T cells makes CD7-directed CAR-T cells prone to fratricide during manufacturing. Successful production therefore often requires gene editing or enrichment of naturally CD7-negative T cell subsets. CD97 presents a similar challenge (45). These approaches are feasible, but they increase manufacturing complexity, cost, and translational difficulty (46). In such cases, the major barrier is not only target biology, but also product engineering.

NKG2D ligands are another attractive concept. These stress-induced molecules are upregulated by DNA damage and malignant transformation and are usually absent or low in normal tissues, suggesting a potentially safer therapeutic window (47, 48). Nevertheless, their inducible and heterogeneous expression often limits the efficacy of monotherapy, and they are currently considered more suitable for combination approaches (49).

IL1RAP is also of interest because it contributes to inflammatory cytokine signaling and aberrant FLT3 and c-Kit signaling, thereby supporting AML cell survival and proliferation (50). Its overexpression on AML cells and LSCs, together with minimal expression on normal HSCs, has shown preclinical anti-leukemic activity, but clinical data remain lacking (51). Thus, while biologically intriguing, these emerging targets remain largely at preclinical or early-phase exploratory stages, with their ultimate clinical utility dependent on resolving the specific manufacturing or biological trade-offs they impose.

Taken together, from a clinical-translational standpoint, CLL-1 is the most clinically mature single target, though durability remains a concern; CD33 and CD123 are well-explored but toxicity-prone, best reserved for controlled or combination settings; emerging targets, while expanding the antigenic repertoire, are not yet ready for broad standalone application. Future progress thus depends not merely on novel target discovery, but on strategically matching each target’s profile with the appropriate engineering platform and clinical context.

### 3.2 Heterogeneity as a barrier to durable CAR-T responses in AML

**Figure 2:**
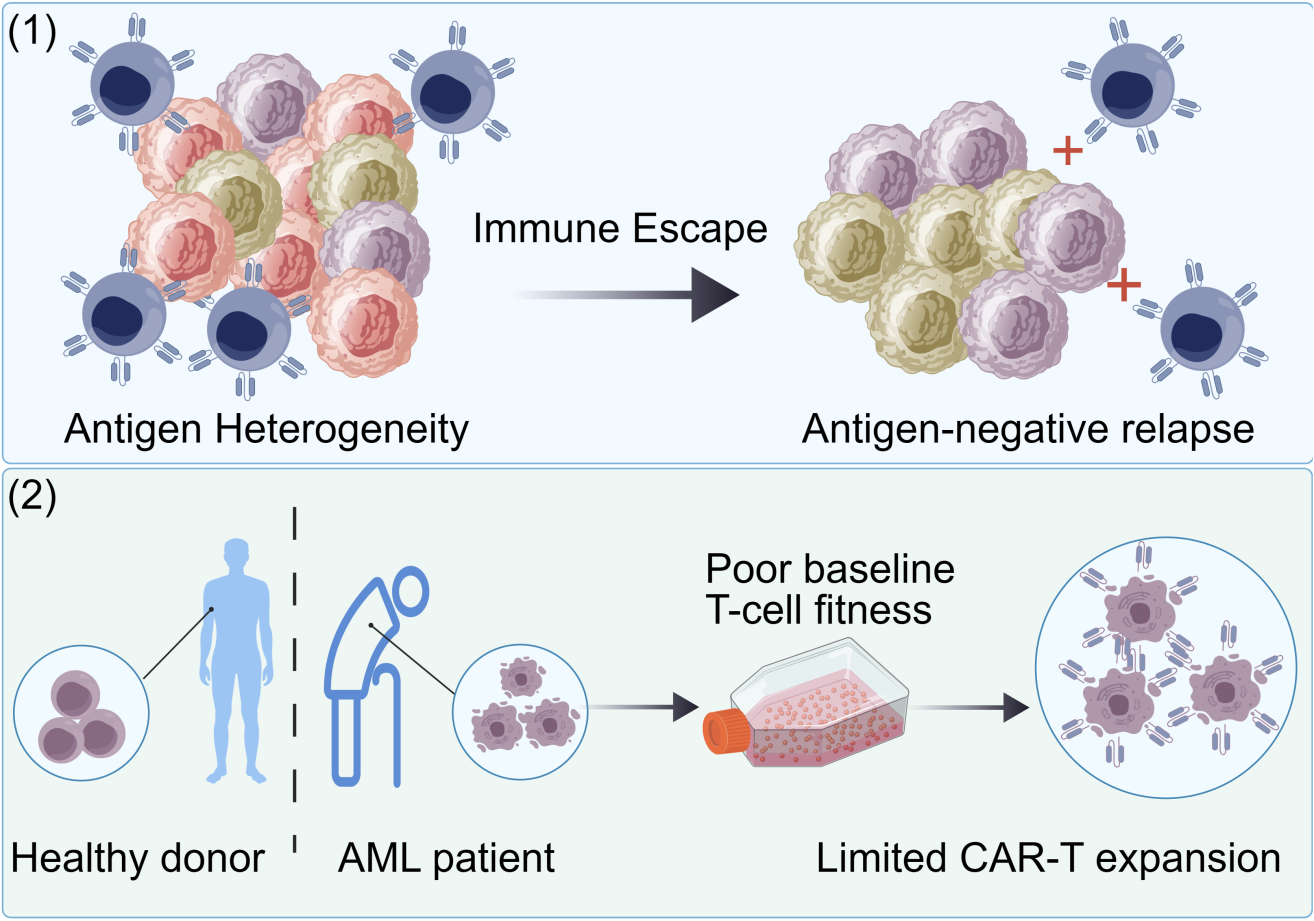
Antigen heterogeneity and impaired T-cell fitness as barriers to CAR-T cell therapy in AML. (1) Antigen heterogeneity in AML creates selective pressure during CAR-T treatment, favoring the outgrowth of antigen-negative leukemic cells and resulting in immune escape and relapse. (2) Compared with healthy donor-derived T cells, AML patient-derived T cells often exhibit reduced baseline fitness, which may impair CAR-T cell production and limit expansion.

AML is a highly heterogeneous disease at the genetic, molecular, and clonal levels, and this heterogeneity represents a major barrier to broad and durable responses after CAR-T cell therapy (52). Importantly, the problem is not confined to the leukemia clone itself. In AML, heterogeneity also extends to disease stage, prior treatment exposure, and the baseline quality of patient-derived T cells, thereby shaping not only target availability but also the functional competence of the final CAR-T product (**Figure 2**). Recent advances in single-cell sequencing, spatial transcriptomics, proteomics, surfaceome profiling, and integrative multi-omics analyses have further highlighted the complexity of AML ecosystems and provided new opportunities for precision antigen selection and rational CAR-T design.

#### 3.2.1 Tumor heterogeneity, clonal evolution, and immune escape

At the genetic and molecular level, AML can be classified into multiple molecular subtypes with distinct driver mutation profiles and clinical outcomes according to classification systems such as those proposed by the European Leukemia Net (ELN). These include, but are not limited to, FLT3-ITD, NPM1, and TP53 mutations (53, 54). Such intrinsic genetic differences directly shape the diversity of antigen expression patterns on leukemic cells, rendering CAR-T products designed against a single target antigen insufficient to cover the full spectrum of AML patients. More importantly, many mutations closely associated with adverse prognosis and therapeutic resistance, including TP53, ASXL1, and RUNX1, do not encode targetable cell surface molecules, thereby posing substantial challenges for CAR-T target selection and product design in high-risk AML subsets (55). Consequently, the patients most in need of effective cellular therapy may also be the least well served by conventional antigen-selection strategies.

Clonal evolution further deepens this limitation. AML is not a static disease, and antigen expression may shift under therapeutic pressure (56). During treatment, newly emerging clones driven by epigenetic silencing or additional genetic alterations may gain a selective growth advantage following the elimination of antigen-sensitive populations, ultimately leading to antigen-negative relapse (57). Thus, target selection based on a single pre-treatment snapshot may be insufficient for long-term disease control.

Recent studies using single-cell RNA sequencing and spatial transcriptomics have provided a more detailed view of AML heterogeneity beyond conventional bulk analyses (58). Within the same leukemic clone, cells can adopt distinct states and show variable antigen expression, which may allow immune escape under therapeutic pressure. Spatial transcriptomic studies further suggest that localized suppressive niches within the marrow may contribute to residual disease and treatment resistance (59). Notably, single-cell atlas studies indicate that no currently available antigen completely separates AML cells from normal hematopoiesis and have identified previously underappreciated targets, including CSF1R and CD86 (60). These observations highlight the dynamic nature of AML and underscore the need for more adaptive and individualized targeting strategies.

#### 3.2.2 Product heterogeneity and intrinsic T cell dysfunction

Another important source of heterogeneity in AML CAR-T therapy is the starting cellular product. In patients with heavily pretreated AML, the T cell compartment is often already shaped by prolonged antigen exposure and prior cytotoxic therapy. Studies have shown that, compared with cells derived from healthy donors, patient-derived CAR-T cells display distinct transcriptional profiles, marked by reduced expression of genes related to T cell activation and long-term memory formation, together with activation of exhaustion-associated programs involving TOX, NR4A, BATF, and IRF4 (61–63). These alterations may limit proliferative capacity and contribute to poor long-term persistence.

Increasing evidence also highlights mitochondrial fitness as an important determinant of CAR-T cell persistence (64). Defective oxidative phosphorylation, excessive reactive oxygen species production, and reduced metabolic flexibility may gradually compromise effector function (65). In parallel, alterations in cellular metabolism can influence epigenetic programs through metabolites such as acetyl-CoA, α-ketoglutarate, and NAD+, thereby stabilizing dysfunctional states that are difficult to reverse (66). Collectively, these findings indicate that variability in CAR-T cell efficacy reflects not only differences in antigen expression and leukemic biology, but also intrinsic properties of the engineered T cells themselves.

### 3.3 The suppressive marrow niche as a barrier to CAR-T function

**Figure 3:**
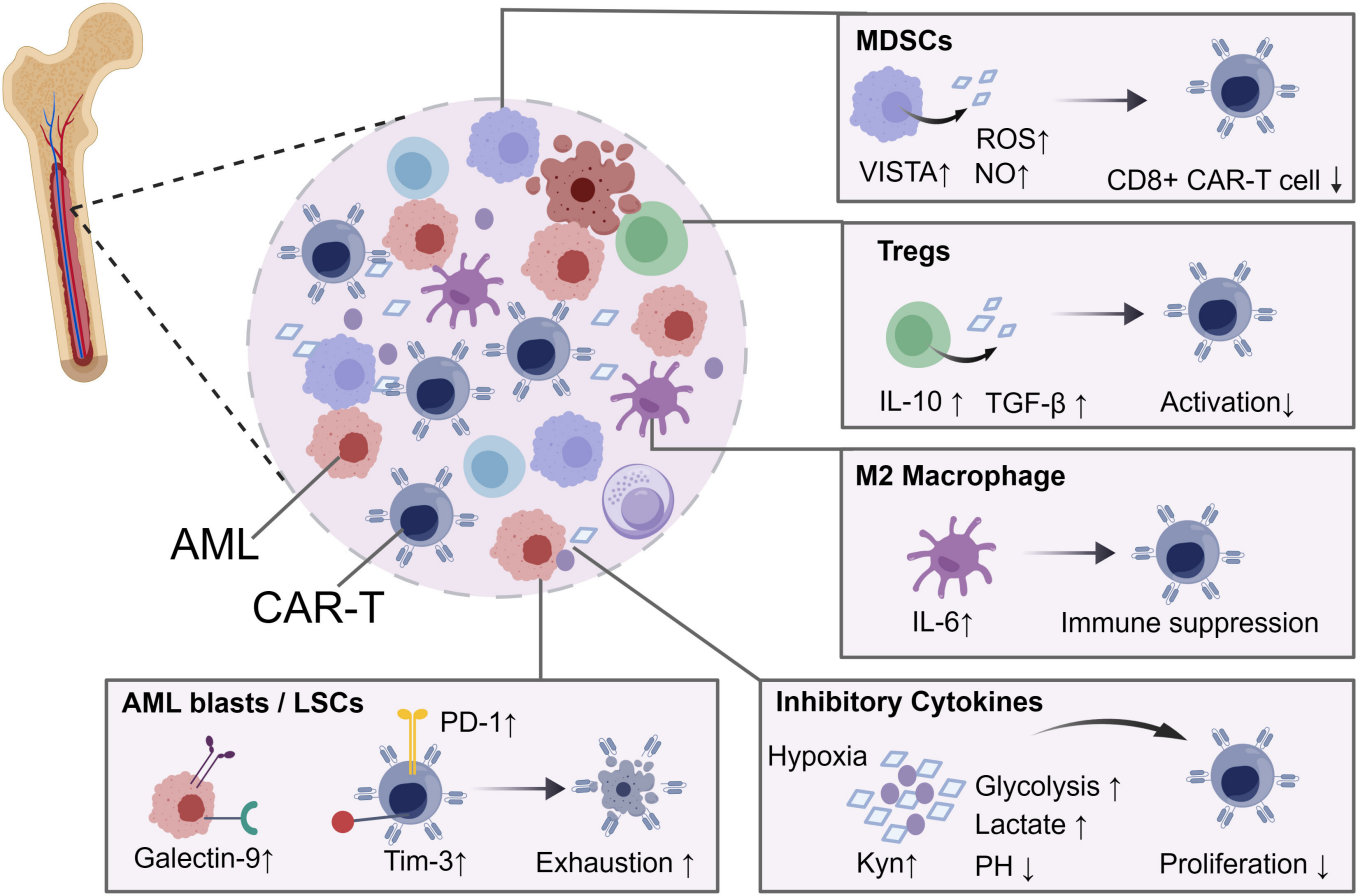
Bone marrow microenvironmental barriers to CAR-T cell therapy in AML. Multiple components of the AML bone marrow microenvironment constrain CAR-T cell activity, including AML blasts/LSCs, MDSCs, Tregs, M2 macrophages, inhibitory cytokines, and metabolically hostile conditions. Together, these factors promote CAR-T cell exhaustion, reduce activation and proliferation, and ultimately weaken antileukemic responses.

The efficacy of CAR-T cell therapy in AML is determined not only by target selection and intrinsic T cell fitness, but also by the profoundly suppressive bone marrow microenvironment (TME). This niche is composed of multiple suppressive immune cell populations, inhibitory soluble factors, and aberrant metabolic conditions that together impair CAR-T cell trafficking, activation, expansion, and long-term persistence. Rather than acting through a single dominant mechanism, the AML marrow microenvironment imposes layered suppression (**Figure 3****).**

#### 3.3.1 Suppressive cellular components in the marrow niche

Within the AML bone marrow microenvironment, multiple cellular components directly or indirectly suppress CAR-T cell responses, including AML blasts and LSCs, myeloid-derived suppressor cells (MDSCs), regulatory T cells (Tregs), and tumor-associated macrophages (TAMs) (67, 68).

AML blasts and LSCs actively contribute to immune evasion. They can reduce their immunogenicity, thereby weakening immune recognition (69). At the same time, they upregulate inhibitory ligands such as galectin-9 and programmed death-ligand 1 (PD-L1), which engage receptors including TIM-3 and PD-1 on CAR-T cells and promote functional exhaustion (70–72). In addition, AML cells frequently exhibit enhanced glycolytic activity, leading to lactate accumulation and an acidic microenvironment. This metabolic shift broadly suppresses T cell function while promoting the recruitment and expansion of MDSCs and Tregs (73).

MDSCs are markedly enriched in the bone marrow of AML patients, where they function as a key immunosuppressive component of the microenvironment (74). These cells inhibit T cell metabolism and proliferation by depleting arginine and tryptophan and by producing reactive oxygen species and nitric oxide (75). Moreover, MDSCs express inhibitory molecules such as VISTA, further dampening CD8^+^ T cell responses (76, 77). Notably, MDSCs also express CD33, suggesting that CD33-targeted CAR-T strategies may exert dual immunomodulatory effects by directly eliminating AML cells while simultaneously depleting key suppressive myeloid populations (78, 79).

Tregs are abnormally expanded in the AML microenvironment and suppress effector T cells and CAR-T cells through both contact-dependent mechanisms and the secretion of inhibitory cytokines such as TGF-β and IL-10 (80). AML cells further support Treg induction and stabilization through molecules such as ICOS ligand and indoleamine 2, 3-dioxygenase (IDO) (81, 82). These observations suggest that Tregs are not merely bystanders, but active participants in maintaining immune tolerance within the marrow niche (83).

Macrophages add another layer of suppression. In AML, they are frequently polarized toward an M2-like phenotype and can inhibit CAR-T cell activity through several mechanisms (84, 85). At the same time, activated macrophages are a major source of interleukin-6 during cytokine release syndrome, underscoring their dual relevance to both therapeutic resistance and treatment-related toxicity (86).

#### 3.3.2 Soluble and metabolic suppression in the marrow niche

Beyond direct cellular interactions, soluble mediators and metabolic constraints within the AML TME further suppress the immunotherapeutic efficacy of CAR-T cells (87, 88). Among these, TGF-β and IL-10 play central roles by directly inhibiting effector function and T cell activation and by expanding regulatory immune populations, thereby reinforcing local immune tolerance (89).

Metabolic dysregulation constitutes another important barrier. Hypoxia-driven enhancement of glycolytic metabolism leads to lactate buildup and microenvironmental acidification, thereby impairing CAR-T cell metabolism, and effector function through suppression of key signaling pathways such as mTOR (63, 90–92). Concurrently, aberrant activation of the tryptophan-catabolizing enzyme IDO1 results in increased production of kynurenine, a metabolic change that promotes T cell dysfunction and favors Treg differentiation and maintenance (93–95). In addition, excessive arginine consumption by MDSCs and other suppressive cells directly restricts CAR-T cell clonal expansion and functional capacity.

Importantly, therapeutic interventions targeting these metabolic pathways are increasingly recognized for their potential synergistic effects. Preclinical studies have demonstrated that modulation of arginine metabolism can significantly enhance the antitumor activity of CD33 CAR-T cells in AML models (96, 97). Furthermore, mutant IDH2 can disrupt glucose metabolism through interference with the pentose phosphate pathway, thereby impairing the metabolic adaptability of CAR-T cells under oxidative stress conditions (98). Although these findings remain largely preclinical, they support the view that effective CAR-T therapy in AML will likely require not only better targeting, but also active remodeling of the metabolic and immunologic constraints imposed by the marrow niche.

## 4 Engineering strategies to overcome AML-specific immune barriers

**Figure 4:**
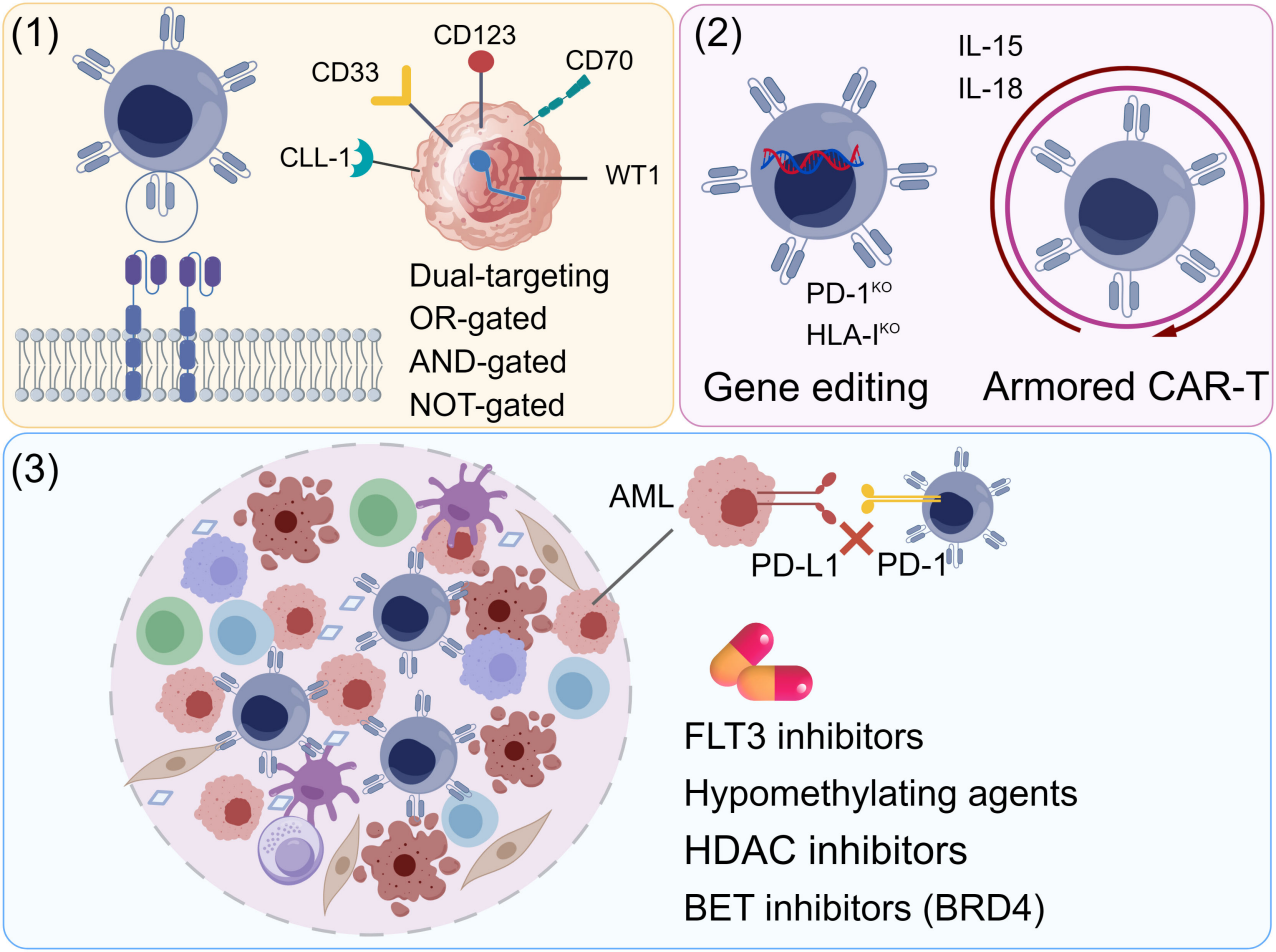
Engineering strategies to overcome key barriers to CAR-T cell therapy in AML. (1) Targeting strategies include the exploration of alternative AML-associated antigens and logic-gated or dual-target CAR designs. (2) CAR-T optimization strategies include gene editing and armored CAR-T cell engineering. (3) Microenvironment remodeling strategies include checkpoint inhibition and combination treatments that may alleviate immune suppression in the AML bone marrow niche.

Multiple immune barriers constrain the application of CAR-T therapy in AML and have shaped the direction of current research and development. Accordingly, ongoing strategies focus not only on refining antigen recognition, but also on preserving CAR-T cell functional stability and alleviating the immune and metabolic constraints imposed by the bone marrow microenvironment. From a translational perspective, these approaches fall into three tiers of clinical readiness: dual-targeting and checkpoint blockade are the most clinically advanced; cytokine-armored and transcriptionally engineered CAR-T cells have early clinical signals but need confirmation; logic-gated CARs, adaptor platforms, and metabolic interventions remain largely preclinical (**Figure 4**).

### 4.1 Antigen targeting and recognition optimization

Although numerous antigens have been explored in AML, the limitations imposed by antigen overlap and disease heterogeneity have made single-target approaches vulnerable to relapse and immune escape. Consequently, increasing attention has shifted toward strategies that improve targeting precision and broaden leukemic coverage.

#### 4.1.1 Dual-target and logic-gated CARs

Among current approaches, dual-target strategies appear particularly attractive because they directly address the limitations of single-antigen designs. Such platforms include dual CARs, tandem CARs, and bicistronic or bispecific CAR constructs. In tandem CARs, two antigen-binding domains are incorporated into a single CAR molecule, whereas dual CAR systems express two separate CAR constructs within the same T cell, allowing activation through either or both targets. Combinations such as CD123 with CD33 or CD123 with CLL-1 can partially mitigate immune escape driven by antigen loss or clonal evolution and are currently considered among the most clinically translatable approaches. More sophisticated logic-gated systems remain largely at the preclinical stage. OR-gate CARs permit activation through either target antigen and may better maintain antitumor activity despite heterogeneous antigen expression and antigen loss (99). By comparison, AND-gated systems require defined antigen combinations for full activation. NOT-gated approaches incorporate inhibitory modules that suppress CAR-T activation in the presence of antigens associated with normal tissues (42, 100–102). Although these designs may reduce off-tumor toxicity, most remain at the preclinical stage because of their greater engineering complexity and limited clinical experience.

Adaptor CAR systems provide another flexible approach. In these platforms, CAR-T cells recognize a universal adaptor molecule rather than the tumor antigen itself, allowing target specificity to be redirected through soluble adaptor antibodies (103). However, adaptor CARs are still largely confined to preclinical and early translational studies.

#### 4.1.2 Alternative recognition platforms

Efforts to expand the target space in AML have increasingly focused on antigens beyond conventional lineage-associated markers. One emerging focus involves tumor-specific post-translationally modified antigens. AML cells often display aberrant glycosylation patterns, including overexpression or structural alterations of sialylated glycans and Lewis Y antigens (58, 104–106). These antigens may provide more selective recognition opportunities because of their relatively low expression on normal HSPCs.

Another direction involves aberrantly overexpressed intracellular antigens, such as WT1 and PRAME. Although these targets are not directly accessible to conventional CAR constructs, alternative MHC-restricted recognition platforms, including TCR-like antibodies, may broaden the spectrum of actionable antigens (107, 108). In parallel, advances in genomics, transcriptomics, proteomics, and surfaceome analyses are increasingly being integrated to facilitate antigen discovery. These multidimensional approaches facilitate the identification of candidate antigens that are stably expressed in defined AML subsets and have minimal impact on normal hematopoiesis, thereby supporting patient stratification and personalized CAR-T cell design (105, 109–112).

### 4.2 Enhancing the persistence and functional fitness of CAR-T cells

Beyond limitations in antigen recognition, the efficacy of CAR-T therapy in AML is also constrained by the compromised baseline state of patient-derived T cells. Accordingly, researchers have increasingly turned to engineering strategies to improve CAR-T cell persistence, functional fitness, and adaptability, particularly in the context of heterogeneous starting products and intrinsically compromised AML -derived T cells.

The choice of costimulatory signaling domains plays a decisive role in determining CAR-T cell persistence. Distinct costimulatory motifs, such as CD28 and 4-1BB, differ substantially in signaling strength, duration, and associated metabolic reprogramming, thereby influencing the balance between effector differentiation and memory formation in CAR-T cells (113). Rational modulation of costimulatory signaling is therefore considered a key design principle for balancing potent initial cytotoxicity with sustained long-term functionality.

The development of so-called “armored” CAR-T cells capable of sensing the TME and dynamically regulating their own functional state has emerged as another important strategy to enhance persistence. In addition to early work with IL-12, cytokines such as IL-15, IL-18, and IL-21 have attracted increasing interest in AML. In preclinical AML models, IL-21/IL-21R signaling may promote differentiation of LSCs, thereby enhancing their susceptibility to CAR-T cell killing (114). Similarly, enforced IL-15 expression in CD64-directed CAR-T cells has been associated with enhanced memory-related transcriptional programs, improved *in vivo* expansion, and prolonged functional persistence in AML models (115). Early clinical data also suggest that IL-18-secreting CLL-1 CAR-T cells may achieve meaningful *in vivo* expansion and leukemia clearance even at relatively low doses (116). Although most evidence remains limited to preclinical or early-phase studies, cytokine-armored CAR-T cells currently represent one of the most clinically translatable approaches.

Transcriptional engineering represents another promising direction for improving CAR-T cell fitness. For example, enforced expression of c-JUN has been shown to restore signaling pathways impaired in the AML marrow environment, enhance costimulatory molecule expression and effector cytokine production, and partially recover proliferative and cytotoxic capacity (117). Among persistence-enhancing strategies, cytokine-armored CAR-T cells have the strongest near-term clinical potential, costimulatory optimization is a mature standard, and transcriptional reprogramming remains experimental.

### 4.3 Remodeling the bone marrow microenvironment

In AML, multiple immunosuppressive factors constrain CAR-T cell expansion and persistence. Accordingly, current efforts have focused on relieving immune checkpoint mediated inhibition, targeting immunosuppressive cellular populations, and modulating the bone marrow immune niche through pharmacologic or metabolic interventions.

Immune checkpoint signaling represents one of the most prominent inhibitory mechanisms in the AML bone marrow microenvironment. To address this barrier, immune checkpoint blockade has been incorporated into CAR-T treatment paradigms, either through combination with checkpoint inhibitors or via genetic disruption of inhibitory receptors within CAR-T cells. For example, silencing PD-1 has been shown to enhance the cytotoxic activity and *in vivo* antileukemic efficacy of CLL-1 CAR-T cells in AML models (118). Among these approaches, PD-1 pathway modulation currently has the strongest translational rationale because of existing clinical experience with checkpoint inhibitors, although long-term safety and the risk of excessive immune activation remain concerns.

Another approach is to reduce the influence of suppressive cellular populations within the marrow microenvironment. In preclinical models, CAR-T cells dual-targeting CD123 and NKG2D ligands were able to eradicate leukemic cells while selectively reducing the impact of immunosuppressive cell populations, thereby indirectly improving the local immune milieu (119). This example suggests that CAR-T cells may be engineered not only for direct leukemia killing, but also to lessen suppressive pressure within the marrow niche.

Pharmacologic combination strategies represent a broader approach to remodeling the marrow microenvironment. Some can directly improve the state of CAR-T cells. Others enhance efficacy by increasing the susceptibility of leukemic cells to immune killing or by partially altering the surrounding microenvironment. For example, BRD4 inhibition has been shown to reduce exhaustion-associated gene expression, limit terminal differentiation, and preserve stem-like memory subsets, thereby supporting more durable antitumor activity (120). Hypomethylating agents and histone deacetylase inhibitors may likewise increase AML cell sensitivity to CAR-T-mediated killing and may also reduce immunosuppressive cell populations within the marrow microenvironment (121, 122). Similarly, in FLT3-ITD^+^ AML, the combination of FLT3-directed CAR-T cells with FLT3 inhibitors has also been reported to improve antileukemic activity.

Metabolic intervention is emerging as another complementary strategy. IDO inhibitors can reduce tryptophan catabolism, whereas lactate dehydrogenase inhibition can limit lactate accumulation. Both approaches may partially restore CAR-T cell survival and function under metabolically unfavorable conditions. Targeting arginine metabolic restriction has also been shown to enhance T cell mediated antitumor immune responses in leukemia models (97). Although these approaches remain largely investigational, combination strategies involving existing agents may represent one of the most clinically feasible directions. In summary, the most practical near-term strategy is combining CAR-T with existing approved agents such as hypomethylating agents and FLT3 inhibitors. By contrast, checkpoint inhibition requires careful toxicity management, and metabolic or other experimental engineering approaches remain too preliminary for current clinical application.

## 5 Key clinical insights from early-phase CAR-T trials in AML

Clinical experience with CAR-T cell therapy in AML remains limited and is derived mainly from early-phase studies conducted in patients with relapsed or refractory disease. Although these trials differ substantially in target antigen, CAR design, cell source, treatment setting, and use of consolidation therapy, a clear hierarchy of clinical maturity across different target antigens and engineering platforms has begun to emerge (**Table 1**). CLL-1 CAR-T represents the most clinically advanced antigen platform, CD33/CD123 approaches remain constrained by toxicity and are primarily used in bridging or consolidation settings, whereas CD70- and CD7-directed or engineered CAR-T platforms remain in early translational stages.

**Table 1:** Recent clinical trials of CAR-T cells for AML.

Targets	Special engineering features	Phase	Patient population	CAR-T dose	Recruitment	Bridge to allo-HSCT	Efficacy outcomes	Toxicity	Cause of failure	Clinical positioning	ClinicalTrials.gov ID	Reference
CD123	CD28ζ; CD20 safety switch	I	Pediatric R/R AML	3×105-3×106 cells/kg	12 enrolled (5 treated)	Allowed in responders	1 CR with relapse; 1 CR off-protocol; others NR	On-target myelosuppression (prolonged cytopenias); endothelial toxicity (capillary leak syndrome).	Limited persistence; cytokine-mediated resistance in TME	Bridge to transplant (preferred); standalone limited by toxicity and poor persistence	NCT04318678	(123)
CD123	Switchable UniCAR; universal CAR-T + CD123 TM	I	Adult R/R AML	UniCAR-T + TM; doses NR	14 enrolled (12 treated)	Not reported	Transient blast reduction; no durable CR reported				NCT02159495	(124)
CD123	universal CAR-T + CD123 TM	1a	Adult R/R AML	1×108-2.5×108 cells/kg	8 enrolled (3 treated)	Not reported	1 PR and 2 CRi; disease control up to 100 days in one patient				NCT04230265	(125)
CD123	anti-CD123 with CD28 and CD3ζ signaling domains	I	Adult R/R AML	DL0-DL1: 5×107-2×108 cells	18 enrolled (7 treated)	Not reported	MLFS/CR observed in DL0-DL1; transient responses and disease stabilization in others				NCT02159495	(126)
CD123	Conventional CD123 CAR-T	I	Adult R/R AML	5×105-2×106 cells/kg	22 enrolled (12 treated)	Not reported	3CR;1CRi;3SD				NCT03766126	(127)
CD33	Nonviral UltraCAR-T; CD33 targeting + mbIL15 + suicide switch	I/Ib	Adult R/R AML	DL1-DL4: 3×104-1×107 cells/kg	15	Allowed in responders	1 CRi, 1 CRh, 1 PR	Severe myelosuppression; prolonged aplasia; hepatic toxicity (SOS); narrow therapeutic window	Prolonged aplasia; on-target/off-tumor toxicity limiting dose escalation	Bridge to transplant (if response); safety-switch or regulated platforms required; standalone not feasible	NCT03927261	(128)
CD33	Conventional CD33 CAR-T	I	Adult R/R AML	3×105 cells/kg	10 enrolled (3 treated)	Not reported	Disease progression leading to death				NCT03126864	(28)
CD33	Drug-regulated CAR; rapamycin-dependent activation	I	Pediatric and young adult R/R AML	1×106 cells/kg	3	Not reported	Drug-dependent disease control with transient blast reduction				NCT05105152	(129)
CD33	Conventional CD33 CAR-T	I/II	Pediatric and young adult R/R AML (≤21 yrs)	DL1-DL4: 3×105-1×107 cells/kg	24 enrolled (19 treated)	Not reported	MRD-CR achieved at DL4; limited activity at lower doses.				NCT03971799	(130)
CD33	Functionally enhanced CD33 CAR-T (human CD33 scFv + enhancing molecules, P2A-linked)	I	Adult R/R AML	5×105 (± 20%) cells/kg	4	Allowed in responders	Allowed in responders				NCT04835519	(131)
CD33	C-JUN over expressing CAR-T cell	I	Over expressing C-JUN R/R AML	0.5×106 (± 20%) cells/kg	4	Allowed in responders	2CRi				NCT04835519	(117)
CLL1	Conventional CLL1 CAR-T	I/II	Adult R/R AML	DL1-DL4: 0.5×106-2×106 cells/kg	38	Allowed in responders	ORR 73.7%; MRD-CR 42.1%; median PFS 9 mo; median OS 12.2 mo; 2-yr OS 51.4%	Myelosuppression (neutrophil/monocyte expression); manageable but persistent	Antigen escape (CLL-1 loss at relapse); limited persistence	Bridge to transplant (most realistic); standalone limited by durability; highest ORR among targets	ChiCTR2000041054	(127)
CLL1	Conventional CLL1 CAR-T	I/II	Pediatric R/R AML	0.35-1×106 cells/kg	8	Allowed in responders	4 had CR and MRD negative; 1 CR with MRD positive; 1 CRi; 1 PR; 1 SD				NCT03222674 ChiCTR1900027684	(132)
CLL1	Conventional CLL1 CAR-T	I	Adult R/R AML	1-2×106 cells/kg	10	Allowed in responders	7 CR/CRi; 2 died due to severe agranulocytosis				ChiCTR2000041054	(29)
CLL1	PD-1 knockdown CLL-1 CAR-T	I	Adult R/R AML	1×107 cells/kg	2	Allowed in responders	CRi				NCT04884984	(133)
CLL1	iCasp9 (FKBP–caspase 9)–based safety switch	I	Pediatric R/R AML	≥1×106 cells/kg	4	Allowed in responders	3 CR				NCT03222674	(134)
CLL1	Allogeneic CRISPR-edited CAR-T (CB-012)	I	Adult R/R AML	No Results Posted	No Results Posted	No Results Posted	Results not yet reported				NCT06128044	(135)
CLL1	Armored CAR-T secreting IL-18	I	Adult R/R AML	3×104 or 3×105 cells/kg	5	Allowed in responders	3 MRD-MLFS				NCT06017258	(117)
CD38	Conventional CD38 CAR-T	I	Adult R/R AML	8.05×105 cells/kg	6	No Results Posted	1 CR 3 CRi	Potential off-tumor toxicity; incomplete LSC coverage	Residual disease; heterogeneous expression	Dual-target combination rather than standalone therapy	NCT04351022	(43)
CD7	CD7 knockout to prevent fratricide; enhanced persistence	I	Adult R/R AML T cell lymphoma T-ALL	DL1-DL3: 1-3×107 cells/kg	12 only 1 with AML	Allowed in responders	7 CR (AML patient had CR)	Fratricide (manufacturing challenge); limited toxicity data in AML	Manufacturing complexity (gene editing/CD7-negative selection); limited AML-specific data	Off-the-shelf allogeneic platform (scalability focus; AML data pending)	NCT04538599	(46)
CD7	Naturally selected CD7-negative CAR-T	I	Adult R/R AML (CD7+)	DL1-DL2: 5×105-1×106 cells/kg	10	Allowed in responders	7 CR;3 NR				NCT04938115	(134)
CD7	Non–gene-edited CD7 CAR-T; CD7 surface expression blocked to prevent fratricide	I	Adult R/R AML (CD7+)	5×106 cells/kg	1	allo-HSCT	CR				NCT04762485	(136)
CD7	Nanobody-based CD7 CAR; naturally selected CD7^-^ CAR-T	I	Adult R/R AML (CD7+)	5×105 or 1×106 cells/kg	10	Allowed in responders	CR achieved in 70% (7/10) of patients				NCT04938115	(137)
NKG2D	Conventional NKG2D CAR-T	I	Adult R/R AML	3×108-3×109 cells/kg	25 enrolled (16 treated, 12 cases were AML)	Allowed in responders	3 objective responses observed	Limited; appears safer (no HSC expression)	Inducible/heterogeneous expression; limited monotherapy efficacy	Combination with microenvironment modulation; not as monotherapy	NCT03018405	(49)
NKG2D	MICA/MICB knockdown; fratricide-free NKG2DL CAR	I	Adult R/R AML or MDS	DL1-DL3: 1×108-1×109 cells/kg	17	No reported	ORR 33% at the recommended dose (DL3)				NCT03466320 EudraCT 2019-001816-46	(138)
CD19	Conventional CD19 CAR-T	II	Adult relapsed CD19+ t (8,21) AML	5-20×106 cells/kg	10	Allowed in responders	CR achieved in 100% (10/10); 60% molecular MRD-CR; median OS 11.6 mo; 12-mo relapse 53.3%	CRS/ICANS (similar to B-ALL profile; manageable).	CD19 expression restricted to rare t(8;21) AML subset	Precision therapy for CD19+t(8;21) AML subset only. Not broadly applicable.	NCT03896854	(139)

CAR-T, chimeric antigen receptor T cell; CR, complete remission; CRi, complete remission with incomplete hematologic recovery; PR, partial remission; SD, stable disease; ORR, objective response rate; PFS, progression-free survival; OS, overall survival; MRD, minimal residual disease; MLFS, morphologic leukemia-free state; CRS, cytokine release syndrome; ICANS, immune effector cell–associated neurotoxicity syndrome; DLT, dose-limiting toxicity; GVHD, graft-versus-host disease.; allo-HSCT, allogeneic hematopoietic stem cell transplantation; mo, months; yr, year.

### 5.1 Clinical maturity of targets: CLL-1 leads

Among currently explored targets, CLL-1 stands out as the most clinically mature. Across multiple early-phase studies, CLL-1-directed CAR-T cells have achieved objective response rates exceeding 70% in some reports, with complete remission rates of approximately 40%-50% in relapsed/refractory AML. These response rates are notably higher than those reported for CD33- or CD123-targeted approaches in similar patient populations. However, durable disease control remains limited, and relapse continues to be the dominant clinical challenge. Subsequent investigations have further explored the use of donor-derived CLL-1 CAR-T cells or the incorporation of CLL-1 CAR-T therapy as a salvage approach after allo-HSCT, with the aim of improving the depth and durability of disease control (29, 127, 134, 140). Overall, these studies suggest that enhancing initial cytotoxicity alone may be insufficient for achieving long-term benefit.

### 5.2 Clinical validation of engineering strategies

Several early-phase studies have explored engineering strategies to improve the feasibility, controllability, and functional fitness of CAR-T therapy in AML. For example, gene-edited CD7, drug-regulated CD33, and cytokine-armored or transcriptionally engineered CAR-T cells have shown early signals of improved expansion or functional fitness (46, 116, 117, 137). Conversely, CD123 CAR-T studies have revealed that cytokine signaling within the AML microenvironment can mediate resistance, limiting durability (141). Together, these findings suggest that early engineering advances may improve short-term activity, but durable benefit in AML will likely depend on overcoming microenvironmental resistance in addition to sustaining CAR-T function.

### 5.3 Toxicity profile and persistent barriers

Early AML CAR-T studies reveal a toxicity profile that extends beyond CRS and ICANS. Prolonged cytopenias, infectious complications, and delayed hematopoietic recovery are major clinical burdens. Three interconnected barriers limit durable efficacy. First, CAR-T persistence is often transient and its loss precedes relapse (116, 127). Second, antigen escape is clinically documented; CLL-1 loss at relapse has been observed, supporting the rationale for dual-targeting strategies (127). Third, microenvironmental resistance is evidenced by cytokine-mediated CAR-T dysfunction in CD123 studies (141). In many clinical protocols, CAR-T therapy has been incorporated as a bridging strategy to allo-HSCT to consolidate remission and address prolonged hematopoietic toxicity. In CLL-1 studies, patients who subsequently underwent transplantation tended to experience more durable disease control than those who did not proceed to transplant (29). This observation, together with the high relapse rates after CAR-T alone and the prolonged myelosuppression associated with CD33/CD123-targeted products, positions CAR-T therapy most realistically as a bridge-to-transplant strategy rather than a curative standalone therapy. In addition, although treatment-related secondary malignancies appear to be rare, continued long-term surveillance remains important to better characterize potential late adverse effects of genetically engineered cell therapies (142).

### 5.4 Implications for future trial design

Future trial design should incorporate pre-specified antigen escape monitoring, mandatory biopsy at relapse, and adaptive designs that allow switching between single-target, dual-target, or logic-gated CARs based on evolving antigen profiles. Combination strategies with hypomethylating agents or targeted inhibitors (e.g., FLT3 inhibitors) should be integrated into early-phase trials rather than tested separately. The development of off-the-shelf allogeneic platforms and combination strategies may further expand the applicability of CAR-T therapy and improve its scalability across broader AML populations. As additional prospective studies emerge, future trial design will need to better define patient selection, the role of combination approaches and transplantation, and the optimal timing of CAR-T therapy within AML treatment paradigms.

## 6 Conclusion

**Figure 5:**
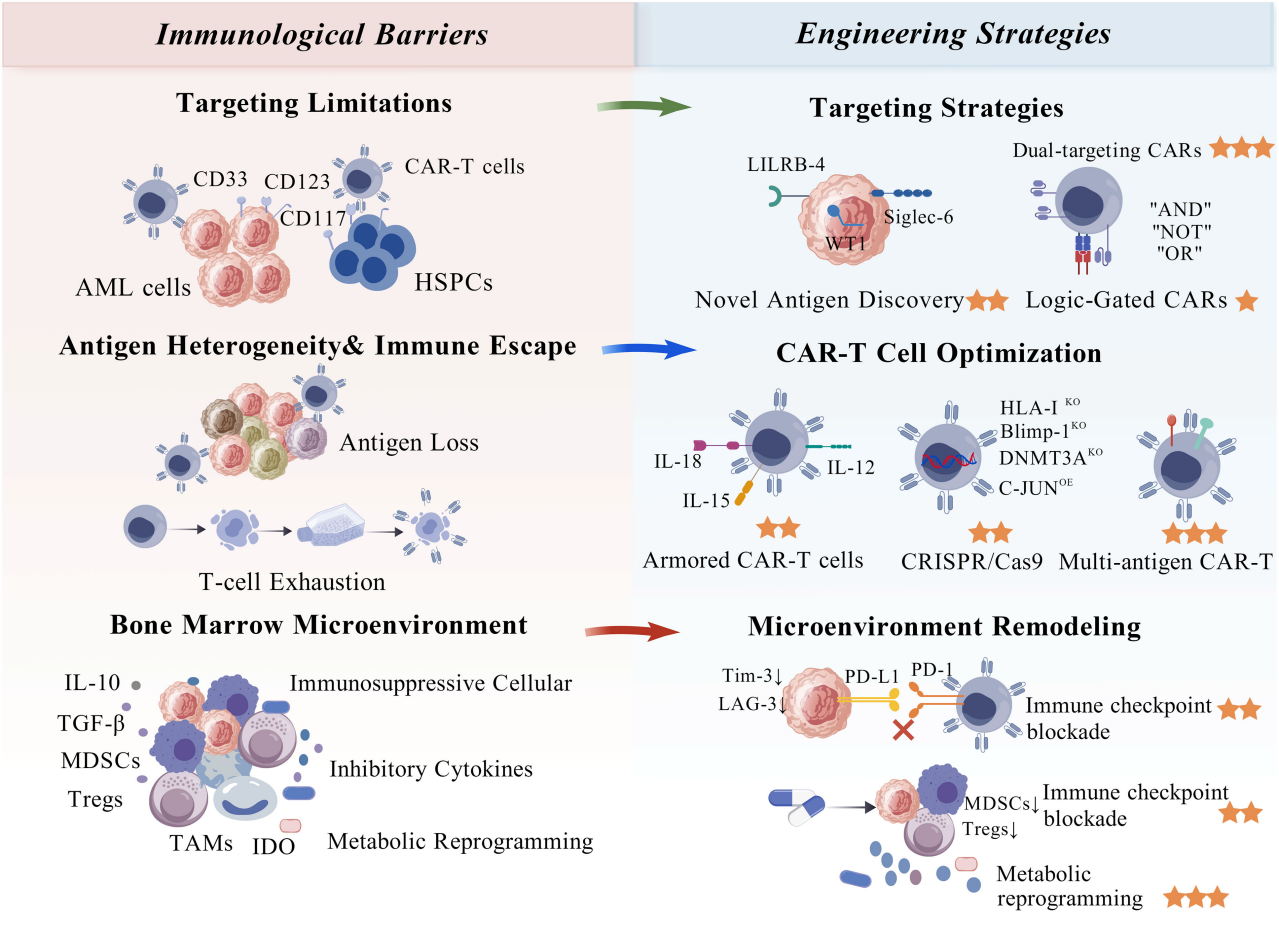
Immunological barriers and engineering strategies for CAR-T cell therapy in AML. The left panel summarizes three major obstacles to effective CAR-T therapy in AML: limited target specificity, antigen heterogeneity with immune escape, and the suppressive bone marrow microenvironment. The right panel illustrates corresponding engineering solutions, including improved antigen-targeting strategies, CAR-T cell optimization, and microenvironment remodeling, aimed at enhancing therapeutic specificity, persistence, and antileukemic efficacy. Star ratings (★) indicate the relative clinical maturity of each engineering strategy.

CAR-T therapy for AML has entered a critical phase where clinical proof-of-concept has been established, but durable responses remain elusive. Several priorities emerge from the evidence reviewed here. First, dual-targeting approaches, particularly combinations involving CLL-1, CD33, or CD123, offer the most direct strategy to counteract antigen heterogeneity and escape, with several constructs already in early-phase testing. Second, enhancing CAR-T persistence through cytokine-armoring (e.g., IL-15 or IL-18) or transcriptional reprogramming (e.g., c-JUN overexpression) represents a clinically feasible direction to address the functional compromise of patient-derived T cells and the hostile marrow environment. Third, safety-controlled platforms, including drug-regulated CARs and logic-gated designs, are essential to manage on-target/off-tumor toxicity. Fourth, combination strategies, especially with hypomethylating agents or FLT3 inhibitors, can be integrated into existing treatment paradigms. Finally, the positioning of CAR-T therapy as a bridge to allogeneic transplantation, rather than a standalone curative strategy, is supported by the frequency of post-CAR-T transplant in responders. Overall, these directions provide a feasible path for transforming AML CAR-T therapy from the early research stage to a therapy with practical clinical significance. The overall relationship between the major biological barriers and the corresponding engineering strategies discussed in this review is summarized in **Figure 5**.

In summary, CAR-T therapy in AML remains promising, but its clinical development continues to face substantial biological and translational barriers. Further progress will depend on a better understanding of AML immunobiology, improved control of CAR-T cell function, and more effective strategies to overcome the suppressive bone marrow microenvironment. Ultimately, CAR-T cell therapy is likely to become an important component of personalized and combination-based treatment strategies rather than a stand-alone solution for AML.
