# Supplementary figures and images for: Immunological barriers and engineering strategies for CAR-T cell therapy in acute myeloid leukemia
Source: Front Immunol. 2026 Jul 15;17:1837609. doi: 10.3389/fimmu.2026.1837609 (PMC13415586; doi:10.3389/fimmu.2026.1837609)

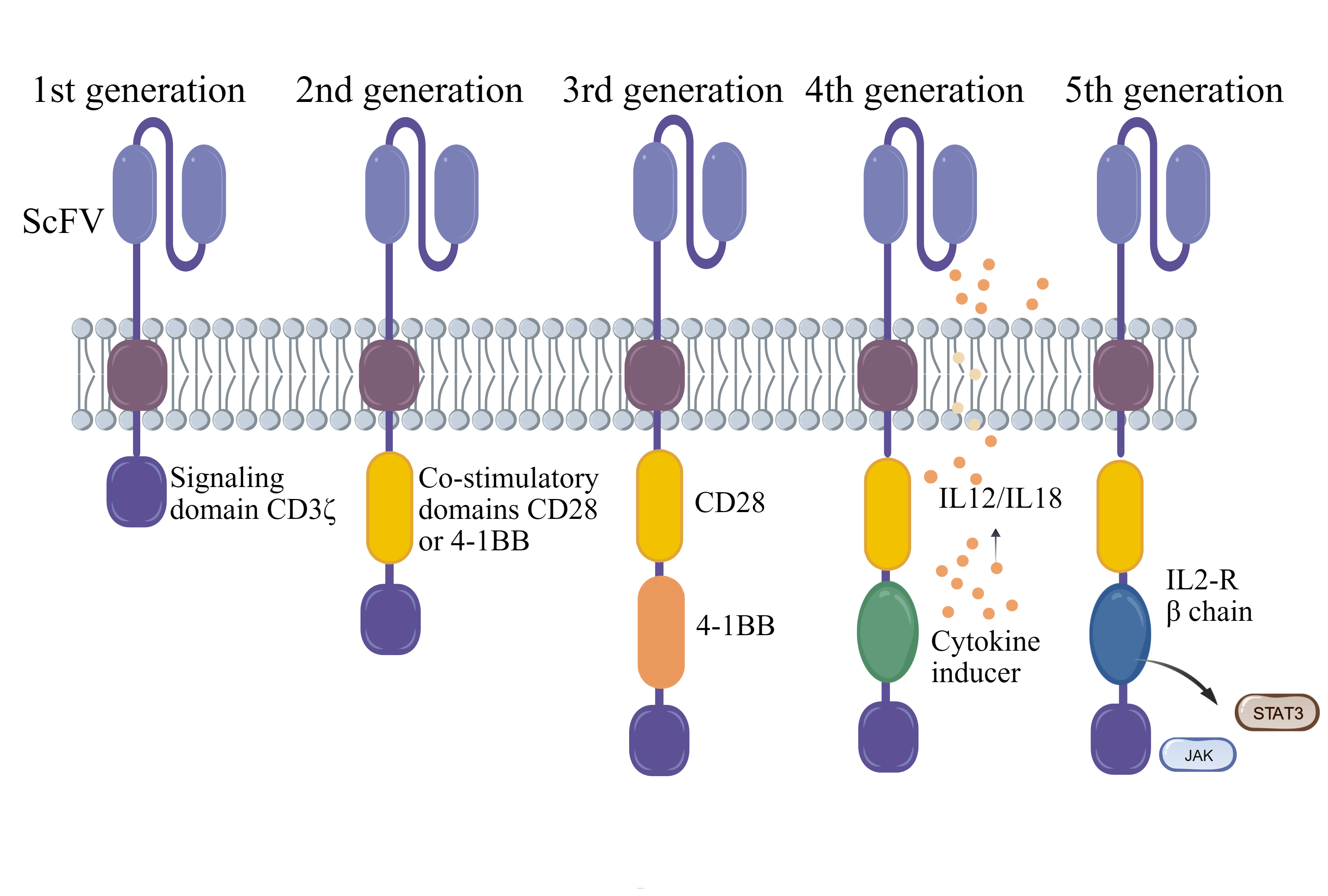

Supplement: Supplementary file 1 [file Image1.tiff]
